# The mental health status of refugees and asylum seekers attending a refugee health clinic including comparisons with a matched sample of Australian-born residents

**DOI:** 10.1186/s12888-017-1239-9

**Published:** 2017-02-21

**Authors:** Frances Shawyer, Joanne C. Enticott, Andrew A. Block, I-Hao Cheng, Graham N. Meadows

**Affiliations:** 1 Southern Synergy, Department of Psychiatry, School of Clinical Sciences at Monash Health, Monash University, Clayton, VIC 3800 Australia; 2 Royal District Nursing Service Institute, 31 Alma Rd, St Kilda, VIC Australia; 3 Special Medicine and Refugee Health & Wellbeing, Monash Health, Dandenong, VIC 3175 Australia; 4 Department of Medicine, School of Clinical Sciences at Monash Health, Monash University, Clayton, VIC 3800 Australia; 5 Southern Academic Primary Care Research Unit, Department of General Practice, Monash University, Notting Hill, VIC 3168 Australia; 6 Mental Health Program, Monash Health, Dandenong, VIC 3075 Australia; 7 Melbourne School of Population and Global Health, University of Melbourne, Parkville, VIC 3010 Australia

**Keywords:** Refugee, Asylum seeker, Epidemiology, Screening, Mental disorders, Posttraumatic stress disorder

## Abstract

**Background:**

The aim of this study was to survey refugees and asylum-seekers attending a Refugee Health Service in Melbourne, Australia to estimate the prevalence of psychiatric disorders based on screening measures and with post-traumatic stress disorder (PTSD) specifically highlighted. A secondary aim was to compare the prevalence findings with Australian-born matched comparators from the 2007 National Survey of Mental Health and Well-Being.

**Methods:**

We conducted a cross-sectional survey of 135 refugees and asylum-seeker participants using instruments including Kessler-10 (K10) and PTSD-8 to obtain estimates of the prevalence of mental disorders. We also performed a comparative analysis using matched sets of one participant and four Australian-born residents, comparing prevalence results with conditional Poisson regression estimated risk ratios (RR).

**Results:**

The prevalence of mental illness as measured by K10 was 50.4%, while 22.9% and 31.3% of participants screened positive for PTSD symptoms in the previous month and lifetime, respectively. The matched analysis yielded a risk ratio of 3.16 [95% confidence interval (CI): 2.30, 4.34] for abnormal K10, 2.25 (95% CI: 1.53, 3.29) for PTSD-lifetime and 4.44 (95% CI: 2.64, 7.48) for PTSD-month.

**Conclusions:**

This information on high absolute and relative risk of mental illness substantiate the increased need for mental health screening and care in this and potentially other refugee clinics and should be considered in relation to service planning. While the results cannot be generalised outside this setting, the method may be more broadly applicable, enabling the rapid collection of key information to support service planning for new waves of refugees and asylum-seekers. Matching data with existing national surveys is a useful way to estimate differences between groups at no additional cost, especially when the target group is comparatively small within a population.

## Background

The year 2014, globally, saw 59.5 million people forcibly displaced, the highest level on record. By the end of 2014, the Syrian Arab Republic became the largest source country of refugees, overtaking Afghanistan, which had held this position for over 30 years. The scale of forced global displacement of people is both unprecedented and accelerating, growing 40% over just 3 years from 42.4 million in 2011 [1]. Some 30 countries, including Australia, have granted or pledged nearly 180,000 places for Syrian refugees since 2013 [2].

Placing large numbers of refugees and asylum-seekers in the community over a short period of time can create particular challenges for services in high settlement areas. Physical and psychological stresses that many refugees and asylum-seekers undergo in their countries of origin, during transition and on arrival in the host country, can increase risks of mental health problems [3], with variable effects across the range of refugee and asylum-seeker populations [4, 5]. To plan appropriate culturally-sensitive treatment, mental health services in areas with high refugee and asylum-seeker populations need to understand the mental health problems of their local client base.

From 2008 to 2013, Australia experienced another period where the numbers of people seeking asylum rapidly increased. Asylum-seekers arriving by boat added to already growing numbers of international students seeking protection [6]. In 2012–2013, 25,091 asylum-seekers arrived by boat [7], up more than 300% on the previous year [8] and the number of places in Australia’s Humanitarian Program increased to 20,019, up from 13,759 in 2011–2012 [7, 9]. Most arrivals were placed in the community either on a protection visa (4,949), allowing for permanent resettlement on the basis that refugee status had been established, or on a bridging visa (16,541) enabling the recipient to remain in Australia as an asylum seeker whilst application for refugee status was determined [7].

The work reported herein took place in a government-funded Refugee Health Service (RHS) located in a high settlement area for refugees and asylum seekers at the peak of this last major influx of new arrivals. Established in 2007 in an urban area of Victoria, Australia, the service had an initial focus on meeting the physical health needs of its clients. In the context of specific concerns raised in clinical work and overstretched local services, the service expanded its brief to address mental health needs. However, despite having an awareness that many refugees and asylum seeker groups may be vulnerable to mental health issues, systematic information about the nature of these problems locally was lacking [10]. With limited project funding, a mental health survey was commissioned in order to seek to address this gap, using brief interviews to maximise response rate and minimise the stress of participation.

This paper reports prevalence estimates for mental health problems amongst RHS clients, derived from screening instruments. Findings regarding refugee mental health can be biased both by the demographic profiles of refugee and asylum-seeker samples, which are untypical of the Australian population [11], and by service context. Ideally, comparators might be drawn from a contemporaneous matched survey of Australian born people seen in a comparable service context. Practically however, this would be a demanding exercise, one beyond the funding limitations of this project; also choosing an appropriate service setting would be challenging given pathways to care for the study population differ from those among established resident Australians. A strategy that allows adjustment for some potential confounders of the study observations and that involves no fieldwork cost is to draw matched control observations from an existing community surveys such as the National Survey of Mental Health and Wellbeing (NSMHWB) which provides information on population prevalence of mental disorders and distress [12]. Matching up to four comparators for each participant provides appreciable benefits to study precision [13, 14].

The aims of this paper are to:

1. Report estimates of overall prevalence of mental disorders including PTSD among RHS clients.
2. Establish matched risk ratios by comparing RHS clientele prevalence findings with a matched Australian-born comparator group from the 2007 NSMHWB.
3. Comment on the acceptability of the set of measures used in the survey for RHS clients as a pilot for use in screening for mental illness in this service and elsewhere [15].

The reporting of this study conforms to the Strengthening the Reporting of Observational Studies in Epidemiology (STROBE) guidelines [16].

## Methods

### Design

As previously published [15], there are two main elements to the study design. We quantified mental health in refugees and asylum-seekers attending the RHS then compared results to a matched Australian-born comparator group extracted from the 2007 NSMHWB.

### Setting

The setting is the catchment of Monash Health, the largest public health care provider in the state of Victoria, Australia, providing services to a core population of over 950,000 in the south-eastern suburbs of Melbourne and to a greater population of 1.344 million [17]. The catchment includes Greater Dandenong, which is both the most disadvantaged and culturally diverse municipality in Victoria [18] and it contains disproportionally high numbers of refugees and asylum-seekers. For many years, this region has received the largest percentage of newly arrived refugees in Victoria [19, 20]: 28% of refugees and asylum-seekers in Victoria live in the catchment [19].

The site for recruitment was the RHS. The RHS comprises two sites: a weekly hospital-based outpatient clinic and a clinic based in a community health centre. Recruitment took place in the community health site during 2013 with interviews conducted between April and November. The study was approved by the local governing university and hospital ethics committees [15].

### Participants

Eligible participants could be aged 18–85 years (although what eventuated was a recruited sample aged 18–66 years, see later) and were refugees or asylum-seekers who were attending the RHS community health site for any reason (i.e., physical and/or mental health issues). Because measures were translated in advance to support consistency of interpreting in the research interviews, participants were required to be fluent in either English or at least one of the major languages from the regions of Afghanistan, Sri Lanka or Iran, including Dari/Dari-Hazaragi, Pashto, Persian/Farsi and Tamil. Written informed consent was obtained from all participants.

Control participant data from the Australian-born sample in the NSMHWB Confidentialised Unit Record File (CURF) [21] involved no additional contact with these survey participants. The CURF is made available to approved researchers by the data custodians at the Australian Bureau of Statistics.

### Measures

#### RHS Sample

##### Psychological distress

the Kessler Psychological Distress Scale (K10) [22] is a 10-item self-report measure of psychological distress in the past 30 days which also gives some indication of likelihood of having a current or recent mental disorder. The bands applied in the NSMHWB for likelihood of mental disorder included low (10–15), moderate (16–21), high (22–29) and very high (30–50) [23]. The K10 is also widely-used as a clinical tool by Australian general practitioners (GPs) and other clinicians. The clinical K10 cut-off scores for having distress consistent with an anxiety or depressive disorder is considered as ≥ 20 with the range of scores for levels of clinical severity being mild: 20–24; moderate: 25–29; and severe: 30–50 [24]. K10 scores at or above the clinical cut-off score can be further broken down into anxiety-dominated disorder (K10-anxiety items: 2, 3, 5, 6), depression-dominated disorder (K10-depression items: 1, 4, 7, 8, 9, 10), or a mixed disorder. Anxiety-dominant here is defined as K10-anxiety ≥ 8 and K10-depression < 12; depression dominant as K10-depression ≥ 12 and K10-anxiety < 8; and mixed disorder as K10-anxiety ≥ 8 and K10 depression ≥ 12. While validity of the K10 has not been formally established in refugee populations, it has shown good reliability (α = 0.86) and ease of use in pre-literate Afghan and Kurdish refugees recruited in Australia and New Zealand [25] as well as Iraqi refugees in Australia (α = 0.94) [26].

##### PTSD

Participants read or had read to them a list of traumatic events and asked to answer yes or no as to whether they, or someone close to them such as a family member, had ever experienced or witnessed any of these events, as per criteria from the *Diagnostic and Statistical Manual of Mental Disorders* (4^th^ ed., text rev.; DSM-IV-TR) [27]. The list combined the 17 items in the original Harvard Trauma Questionnaire (HTQ - [28]), with the 11 items from the PTSD section of the Composite International Diagnostic Interview 2.1 [29]; as there was overlap on 6 items, the traumatic events list had 22 items.

Participants who indicated that they had been exposed to a traumatic event were then administered the PTSD-8 [30], an 8-item screening questionnaire derived from the HTQ [28] that assesses the three symptom clusters for DSM-IV PTSD diagnosis. Items for the PTSD-8 are scored on a four point Likert scale, ranging from 1 (‘not at all’) to 4 (‘extremely’). Screening criteria for PTSD are met if at least one item in each symptom cluster has a score of ≥ 3. The PTSD-8 has acceptable performance compared to the HTQ [30] and good Cronbach’s alpha values across three different samples (0.83–0.85) see [15]. PTSD symptoms were assessed across two time frames: symptoms since the trauma (PTSD-lifetime) and symptoms in the past month (PTSD-month).

### Service utilisation

General health service utilisation was recorded for matching purposes by refugee participant answers (Yes/No) to questions condensed from the 2007 NSMHWB assessment: 1) In the past 12 months, have you seen a general practitioner for your own physical or mental health? 2) In the past 12 months, have you been admitted overnight or longer in any hospital for a physical health problem? 3) In the past 12 months, have you seen any kind of specialist health care provider such as a specialist doctor, psychiatrist, psychologist, social worker or anyone else?

Relevant demographic and healthcare use data were also collected [15]. At the end of the interview, participants were asked to rate how acceptable they found the interview on a fully-anchored 7-point scale ranging from 1 = *totally unacceptable* to 7 = *perfectly acceptable*. Measures were interview-administered by trained research assistants using interpreters as required [15].

### NSMHWB Sample

The K10 was also used for the NSMHWB sample along with more detailed but parallel service utilisation questions. PTSD was identified using the World Mental Health Survey Initiative version of the Composite International Diagnostic Interview (WHO-CIDI 3.0; ICD-10 criteria) [31].

### Primary and secondary outcome measures

The K10 is the primary outcome measure, given that the same measure was used in both samples: refugees at the RHS and Australian-born health service users in the community sample [15]. PTSD is the secondary outcome: in the RHS sample it was measured using the PTSD-8 and in the community sample it was identified using the WHO-CIDI 3.0 [31].

### Sample size

The sample size required was calculated to be 130 refugee participants. This is adequate to detect a difference in proportion affected by a mental health condition of 0.125, assuming the proportion affected in the Australian-born NSMHWB sample is 0.25, with power of 0.8, alpha of 0.05 and four matched Australian-born residents per refugee.

### Translation and field testing

To support administration in interview and work towards standardisation of translation by interpreters, all measures were translated into Dari, Pashto, Farsi and Tamil then back-translated into English. The translations were reviewed by cultural advisors appointed to the project then field tested. Cultural advisors were two medical doctors, both refugees themselves, with one being Afghan in nationality (fluent in Dari, Pashto and Farsi) and the other Sri Lankan (fluent in Tamil). The field testing process was conducted over 14 weeks by cultural advisors who trialled the measures with three members of each of the non-English speaking groups. Field testing indicated that the questionnaire items were understood by informants with the use of elaborations and additional explanatory statements. Participant Information and Consent Forms were translated into the nominated languages then reviewed by the cultural advisors see [15].

### Matching strategy - NSMHWB comparison group

Each refugee or asylum-seeker participant was matched by random selection without replacement to four Australian-born comparators from the NSMHWB data set; varying the age criterion to plus/minus 5, 7 or 10 years was investigated to find the best matching strategy that could identify four comparators per participant. In addition to age, the matching criteria were:

- Gender: male/female
- General practice visit/consultation in last twelve months:
o Australian-born group: yes/no.
o Refugees and asylum-seekers: most of the clinic participants in this study had been referred here from a routine health triage assessment for newly arrived asylum-seekers that occurred in the last twelve months. On the assumption that the vast majority of refugee and asylum-seeker participants would have had at least two primary care level service consultations (one via Red Cross triage or GP referral, one in the RHS), RHS participants reporting ‘No’ in the survey to consulting a GP in the previous twelve months were matched to Australian-born comparators reporting 2 GP consultations in the previous twelve months. Refugees and asylum-seekers reporting ‘Yes’ to this question were matched to Australian-born comparators reporting 3 or more GP consultations in the previous twelve months.
- Specialist visit/consultation in last twelve months: yes/no.
- Hospitalisation in in last twelve months: yes/no.

### Statistical analysis

Demographic characteristics of the sample were compared to the Monash Health catchment population and to the asylum-seeker population nationally. As a check for possible selection bias, chi-square tests were used to examine whether there were demographic differences between the participants and potentially eligible clients who declined or were not interviewed before the close of recruitment. Cronbach’s alpha was calculated to assess the internal reliability of the K10 and PTSD-8. The occurrence of any missing data is reported and examined further. In this study, missing data was minimal and handled in the analyses using casewise deletion based on availability of complete data for individual variables.

Based on the survey data collected from refugee and asylum-seeker participants, the overall frequency of K10 scores at or above the clinical cut-off was determined then broken down using the K10 clinical and NSMHWB cut-off scores. The overall frequency of PTSD-8 scores at or above the clinical cut-off indicating a likelihood of having PTSD was first calculated, then broken down using the K10 sub-groups.

Matched comparative analysis [32, 33] with NSMHWB controls included conditional Poisson regression to calculate the relative risk (RR) of having a mental disorder using estimated K10 clinical cut-off scores (abnormal K10 ≥ 20) [33]. The conditional Poisson model creates stratum for each matched set and this helps to control for differences in underlying risk that might confound associations with the outcome of interest. In this study, known confounders were age, gender and health service use, thus these were included in the matching. The 4:1 matched set analysis with 135 matched sets (four comparators for each of the 135 participants) was needed to provide sufficient study power (approximately 80%) to detect significant RRs of 2 or greater when the percentage of controls affected was 5% [14, 34].

## Results

### Participants

All eligible clients, including all new referrals during the recruitment period, were approached by RHS bicultural staff either by phone or during a scheduled appointment and invited to speak to the researchers about participating in the study. In total, 267 clients were invited to take part; of these, 207 accepted the invitation, 55 declined and 5 did not meet eligibility criteria. Of the 207 clients who accepted the invitation, 7 did not meet eligibility criteria, 6 participants declined participation and 59 did not proceed to meet with the researchers before the planned closure of recruitment once power considerations were satisfied [15]. The response rate for agreement to participate was 76.1% (194/255); of these, 135 were interviewed. Based on chi-square tests, there were no significant differences on the available demographic details (gender, country of birth, primary language) between the participant group and those who declined participation or did not proceed to interview before recruitment closed, suggesting that sample selection processes were unlikely to be a critical source of bias.

Demographic characteristics are detailed in Table 1. Most of the sample comprised asylum-seekers (82%) and there were more males (93.3%) compared to the general population within the Monash Health catchment (49.3%) [35]. However, the large number of males in this predominately asylum-seeker sample reflects contemporaneous national figures for asylum-seekers (90.4% male) [36]. With an average age of 35 years, the sample tended to be relatively young and was of working age: only 4 participants were 60 years or over and only one participant was over the age of 64 (and was 66). Most participants were from Afghanistan and Sri Lanka with a minority from Pakistan and Iran. During 2012–2013, the period in which most of the participants were seeking asylum, these source countries had a score from the US State Department of either 4 (Iran, Sri Lanka) or 5 (Afghanistan, Pakistan) on the Political Terror Scale, a widely used scale of human-rights practices ranging from 1 to 5 [37]. A score of 4 indicates that large segments of the population are affected by violations in civil and political rights and that violence is common. A score of 5 indicates that the whole population is affected by terror.

**Table 1:** Descriptive statistics for sample demographic variables

Variable	Available *n*	Results
Age, mean years (range)	135	35.0 (18–66)
Sex, *n* (%)	135	
Male		126 (93.3)
Female		9 (6.7)
Visa category, *n* (%)	134	
Bridging (asylum seeker)		109 (81.3)
Refugee, humanitarian or permanent protection		25 (18.7)
Country of birth, *n* (%)	135	
Afghanistan		65 (48.1)
Iran		7 (5.2)
Pakistan		15 (11.1)
Sri Lanka		48 (35.6)
Ethnic group, *n* (%)	135	
Hazara		79 (58.5)
Tamil		47 (34.8)
Other		9 (6.7)
Marital status, *n* (%)	135	
Single		43 (31.9)
Married		88 (65.2)
De facto		1 (0.7)
Separated/widowed		3 (2.2)
Separated from spouse as a result of arrival, *n* (%)	86	76 (88.4)
Children, *n* (%)	134	
Yes		84 (62.2)
Number of children, mean (range)	82	3.2 (1–7)
Separated from children as a result of arrival, *n* (%)	81	73 (90.1)
First language, *n* (%)	135	
Dari		17 (12.6)
English		2 (1.5)
Farsi		7 (5.2)
Hazaragi		60 (44.4)
Tamil		47 (34.8)
Other		2 (1.5)
Need for interpreter, *n* (%)	135	117 (86.7)
Religion, *n* (%)	134	
Christian		13 (9.7)
Hinduism		33 (24.6)
Islam		85 (63.4)
Nil		3 (2.2)
Level of education completed, *n* (%)	135	
None		33 (24.4)
Primary school		53 (39.3)
High school		31 (23.0)
Trade college		7 (5.2)
University		8 (5.9)
Other		3 (2.2)
Current employment in Australia, *n* (%)	134	6 (4.5)
Employment in home country, *n* (%)	134	122 (91)
Months in Australian immigration detention centres, Mean (range)	134	4.1 (0–24)
Months in refugee camps outside Australia, Mean (range)	135	6.0 (0–228)
Months in Australia, Mean (range)	132	11.8 (1.5–46.4)
Access to Medicare, *n* (%)	135	129 (95.6)

### Performance of measures

All 135 participants completed the K10. Four participants did not complete the PTSD-8 due to concerns by the interviewer or participant about participant distress levels, making complete PTSD case data for 131/135 (97.0%). Those participants who did not complete the PTSD-8 had K10 scores of 26–46 (moderate-severe clinical scores). Only one person who screened positive for current PTSD had a normal K10. Notwithstanding the very small sample size for some languages, the K10 and PTSD-8 demonstrated acceptable to excellent internal consistency across language groups (K10: 0.87–0.94; PTSD-month: 0.79–0.90; PTSD-lifetime: 0.73–0.94), with the exception of PTSD-lifetime for Tamil which was 0.68.

Interview acceptability ratings were available from 129 participants (including 3 of the 4 people with missing data for the PTSD-8). From the available data, 96% of participants rated the interview as acceptable or perfectly acceptable (median rating: 7); no one rated the interview as unacceptable.

### Mental health status

Based on K10 clinical cut-off scores, mental illness prevalence in the clinic population was 50.4% (68/135). Most participants (92.5%) reported having experienced a traumatic event with 22.9% (30/131) screening positive for PTSD-month and 31.3% (41/131) for PTSD-lifetime (PTSD-8). The combined prevalence rate of mental illness over the past month was 51.1%. Almost all those screening positive on PTSD-month had abnormal K10 scores (29/30) and 22/30 were in the severe range (30–50). In those with PTSD-lifetime, 33/41 had abnormal K10 and 26/41 were in the severe range. The breakdown of K10 scores by PTSD is shown in Table 2. Abnormal K10 scores were typically mixed with neither depression nor anxiety dominant (Table 3).

**Table 2:** Prevalence of mental disorders in refugees and asylum-seekers by K10 scores and scoring bands

		Clinical cut off for K10		Clinical bands for abnormal K10			NSMHWB bands for K10				Total
		K10 – normal (10–19) % (*n*)	K10 – abnormal (≥20) % (*n*)	K10- mild (20–24) % (*n*)	K10- moderate (25–29) % (*n*)	K10- severe (30–50) % (*n*)	K10- mild (10–15) % (*n*)	K10- moderate (16–21) % (*n*)	K10- high (22–29) % (*n*)	K10- very high (30–50) % (*n*)	
K10		49.6 (67)	50.4 (68)	8.9 (12)	12.6 (17)	28.9 (39)	33.3 (45)	18.5 (25)	20.0 (27)	28.9 (39)	135
PTSD -lifetime	yes	19.5 (8)	80.5 (33)	2.4 (1)	14.6 (6)	63.4 (26)	7.3 (3)	12.2 (5)	17.1 (7)	63.4 (26)	41
	no	65.6 (59)	34.4 (31)	12.2 (11)	10.0 (9)	12.2 (11)	46.7 (42)	21.1 (19)	20.0 (18)	12.2 (11)	90
PTSD -month	yes	3.3 (1)	96.7 (29)	3.3 (1)	20.0 (6)	73.3 (22)	3.3 (1)	0 (0)	23.3 (7)	73.3 (22)	30
	no	65.4 (66)	34.7 (35)	10.9 (11)	8.9 (9)	14.9 (15)	43.6 (44)	23.8 (24)	17.8 (18)	14.9 (15)	101

Note. All participants completed the K10 (*n* = 135), while 131 completed the PTSD-8

**Table 3:** Prevalence of mental disorders in refugees and asylum-seekers attending the clinic

		K10-normal % (*n*)	K10-depression^a^ % (*n*)	K10-anxiety^b^ % (*n*)	K10-mixed^c^ % (*n*)	Total
All (*n* = 135)		49.6 (67)	0.7 (1)	3.7 (5)	45.9 (62)	135
PTSD – lifetime (*n* = 131)	yes	19.5 (8)	0 (0)	0 (0)	80.5 (33)	41
	no	65.6 (59)	1.1 (1)	5.6 (5)	27.8 (25)	90
PTSD – month (*n* = 131)	yes	3.3 (1)	0 (0)	0 (0)	96.7 (29)	30
	no	65.3 (66)	1.0 (1)	5.0 (5)	28.7 (29)	101

Note. All participants completed the K10 (*n* = 135), while 131 completed the PTSD-8. K10 results positive for a mental disorder (K10 ≥ 20) were further broken down into:

^a^Depression dominant measured by K10-depression ≥12 and K10-anxiety <8

^b^Anxiety dominant measured by K10-anxiety ≥ 8 and K10-depression <12

^c^Mixed measured by K10-anxiety ≥ 8 and K10-depression ≥ 12

### Adjustment of relative risk estimated using matched controls

Of all participants in the NSMHWB, 13.25% scored above clinical cut-off on the K10 (1171/8841). Contrasting this with the above finding of a 50.4% rate in the study group gives an unadjusted RR 3.83 (95% CI: 2.31, 3.16) but this comparison, unadjusted for sample demographics (Table 1) and service context, may overstate the difference. Matching comparison data from the NSMHWB as introduced above adjusts for the demographics of the sample and can also, though less precisely, take into account service use. Matched cases were identified using plus/minus 10-year age groupings in addition to other specified criteria. One participant who reported *yes* to hospitalisation but *no* GP or specialist consultation in previous 12 months had no matched comparators so was excluded from the matched analyses reducing the sample size to 134. This matched 134 refugees and asylum-seeker participants with 535 Australian-born residents, including four Australian-born residents for 133/135 participants and three for 1/135.

Table 4 shows the prevalence of mental disorders in the refugee and asylum-seeker and Australian sample and Table 5 shows the conditional risks ratios when comparing the two groups. Conditional risk ratios were all significant (*p* < 0.02) showing higher risks in refugees and asylum-seekers compared to Australian-born comparators.

**Table 4:** Prevalence of mental disorders in refugees and the matched Australian-born group

	K10 -Depression dominant^a^ % (*n*)	K10 - Anxiety Dominant^b^ % (*n*)	K10 –Mixed^c^ % (*n*)	PTSD-month^d^ % (*n*)	PTSD-lifetime^d^ % (*n*)	Missing^e^ % (*n*)	None % (*n*)
Refugee clinic sample (*n* = 134)	0.7 (1)	3.7 (5)	46.3 (62)	22.4 (30)	30.6 (41)	3.0 (4)	44.0 (59)
Australian-born matched sample (*n* = 535)	2.4 (13)	3.2 (17)	10.5 (56)	5.0 (27)	13.8 (74)	-	72.7 (389)

Note. 4 comparators matched to 133 refugees and 3 comparators matched to one refugee. One refugee subject had no found matched comparators and was removed from this analysis. K10 results positive for a mental disorder (K10 > 19) were further broken down into

^a^Depression dominant measured by K10-depression > =12 and K10-anxiety <8

^b^Anxiety dominant measured by K10-anxiety > =8 and K10-depression <12

^c^Mixed measured by K10-anxiety > =8 and K10-depression > =12

^d^PTSD screened in the refugee sample using the PTSD-8 and classified in the Australian-born matched sample using ICD–10 criteria

^e^Unable to complete the PTSD-8 due to distress

**Table 5:** Risk Ratio estimates comparing mental illness in the study refugee sample with matched Australian-born group

	K10 abnormal (≥ 20) % (*n*)	K10 normal (<20) % (*n*)	Conditional RR^a^ (95% CI)
Refugee clinic sample	50.7 (68)	49.3 (66)	3.16 (2.30 to 4.34)
Australian-born matched sample	16.1 (86)	83.9 (449)	
	PTSD – yes % (*n*)	PTSD – no % (*n*)	
PTSD – lifetime^b^			
Refugee clinic sample	30.6 (41)	66.4 (89)	2.25 (1.53 to 3.29)
Australian-born matched sample	13.8 (74)	86.2 (461)	
PTSD – month^b^			
Refugee clinic sample	22.4 (30)	74.6 (100)	4.44 (2.64 to 7.48)
Australian-born matched sample	5.0 (27)	95.0 (508)	
	Abnormal K10 and/or PTSD % (*n*)	Normal K10 and no PTSD % (*n*)	
K10 and/or PTSD – lifetime^b^			
Refugee clinic sample	56.7 (76)	43.3 (58)	2.84 (2.09 to 3.87)
Australian-born matched sample	26.4 (141)	73.6 (394)	
K10 and/or PTSD – month^b^			
Refugee clinic sample	51.5 (69)	48.5 (65)	2.15 (1.62 to 2.85)
Australian-born matched sample	18.1 (97)	81.9 (438)	

Note. There were 134 refugees with matched comparisons included in this analysis. All refugees completed the K10 but 131 completed the PTSD-8

^a^Risk ratio (RR) is estimated from the conditional Poisson regression model, where the ‘conditional’ refers to stratum created for each matched set

^b^PTSD screened in the refugee sample using the PTSD-8 and classified in the Australian-born matched sample using ICD–10 criteria

From Table 5, it can be seen that, compared to the refugee and asylum-seeker sample, more of the Australian sample who met diagnostic criteria for PTSD (based on WHO-CIDI 3.0) had normal K10 with 11 of 27 (40.7%; 95% CI: 24.5, 59.3) with PTSD-month having normal K10 scores and 55/74 (74.3%; 95% CI: 63.4, 82.9%) with PTSD-lifetime having normal K10. The corresponding figures in the refugee and asylum-seeker sample (based on PTSD-8) were 1/30 (3.3%; 95% CI: 0.6, 16.7%) and 6/74 (8.1%; 95% CI: 3.8, 16.6%). The proportion of K10 positive participants who also screened positive for PTSD was considerably higher in the refugee and asylum-seeker group [PTSD-month: 29/64 (45.3%; 95% CI: 33.7, 57.4%; PTSD-lifetime: 33/64 (51.6%; 95% CI: 39.6, 63.4%)] compared to the Australian sample [PTSD-month: 16/86 (18.6%; 95% CI: 11.8, 28.1%); PTSD-lifetime: 19/86 (22.1%; 95% CI: 14.6, 32.0%)]. Considering the K10 as a screener for 1-month PTSD, Positive Predictive Value (PPV) in the two samples varied considerably (45% vs 19%) but despite use of different PTSD instrumentation, the Likelihood Ratio (LR) took fairly similar values in both samples (LR = 3 in the Refugee survey and LR = 4 in the Australian born sample).

## Discussion

### Summary of findings

The aims of this study were to survey clients attending the RHS in order to determine an overall estimate of the prevalence of mental health disorders then to establish matched risk ratios through comparison with a matched Australian-born comparator group drawn from the 2007 NSMHWB. All participants had arrived in Australia from source countries where human rights violations are common. Results showed that around half the surveyed RHS clients screened positive for a mental health condition over the previous month, while nearly a quarter screened positive for PTSD. With adjustment for demographics and, in part, for service use, this studied population had a RR for an elevated K10 of 3.16 compared to the Australian-born comparison group. Tentatively, given difference in instrumentation, we note that findings support the expectation of elevated risk ratios for PTSD in our refugee and asylum-seeker sample, with refugees and asylum-seekers over four times as likely to have PTSD-month and twice as likely to have PTSD-lifetime. Cases of PTSD as a proportion of K10 positive participants was considerably higher in the refugee and asylum-seeker group compared to the Australian sample.

### Limitations and efforts to minimise bias

Use of brief screening questionnaires as here employed might result in misclassification that could inflate prevalence estimates, however it was preferred as it very likely assisted response rates while serving to pilot practical measures for regular use. Study enrollment closure when the intended sample size was achieved left some willing participants not interviewed however the comparison of demographics between non-respondents does not suggest any substantial selection bias.

Considering adjustment by matching, while this has a strong pragmatic rationale, some limitations arise from differences in sampling used for acquiring the two groups used in this study: the refugee group were sampled from a service setting and the reference group were acquired from users of health services within a community sample survey. We had filtered the community sample to participants who were users of health services to an approximately comparable degree to the RHS clients (based on the same assessment measure) and over a similar 12 month time frame. We did this in order to help reduce bias that could be introduced by comparing a clinic sample with a community sample. While there are precedents for this methodology [38, 39], and community and clinic samples may be more similar than is often assumed [40, 41], the sampling differences should be kept in mind when interpreting the findings. Another limitation was that the two samples had non-contemporaneous data with the refugee data collected in 2013 and the national survey data collected in 2007 [15] but the 2007 survey is the most recent fully comparable data available. Other potential differences in the comparison groups include possible differences in understanding terms related to mental illness and control data not being collected at the time of a consultation. The use of different instruments to assess PTSD (PTSD-8 in the clinic sample and WHO-CIDI 3.0 in the NHMHWB) mean these comparisons may be seen as less precise than those involving the K10, nevertheless we suggest these estimates merit reporting given the importance of PTSD in this group. These limitations notwithstanding, we believe the adjusted analyses are useful as an advance over the unadjusted comparison analysis, which however was presented in the results. The unadjusted RR for high K10 scores was substantially higher than the adjusted comparison estimation and this latter more closely reflects a true estimate given that the refugee sample largely constituted males around the age of 35 years.

It might be expected that most individuals with current PTSD would also score in the abnormal range on the K10. However, in the Australian-born sample, 40.7% of the 27 people testing positive for PTSD-month had a normal K10. This seemingly high percentage might be the product of limitations in sensitivity of the K10 [42], as well as the uncertainty associated with a small sample as indicated by the wide confidence interval associated with this finding (95% CI: 24.5, 59.3%): a larger sample might produce a smaller percentage. We may note that in another national survey 14.8% of males and 20.3% of females with PTSD did not meet criteria for another Axis 1 disorder including anxiety disorders and depression, with all disorders assessed using a diagnostic instrument [43]. If we consider the K10 here as a screen for PTSD 1-month occurrence then it may be relevant that for screening tests generally, PPV often varies with baseline population prevalence, and here in the Australian-born sample the prevalence of PTSD is less than a quarter of that in the refugee group studied here. As noted above, the LR was more similar across the two situations, something commonly observed as a general property of screening instrumentation [44].

### Implications for services

The results of this study support the importance of specific planning for mental health service provision in areas of high refugee settlement, which may also be areas of socioeconomic disadvantage. Recent examination of national surveys has shown socio-economically disadvantaged areas to have significant effects on prevalence of elevated K10 scores [45]. The RR in this examined data for having a K10 score of ≥ 20 in the most disadvantaged group compared to the least disadvantaged group was 2.1 nationally [45]. This finding may reflect the combinations of social drift, occupational and environmental adversity and relative under-treatment in disadvantaged areas [46]. The level of distress in the RHS clinic population was more than three times the matched Australian-born sample. This was greater than to be expected from socio-economic disadvantage alone and therefore, likely causally associated with the high levels of exposure to traumatic events, stressful migration experiences, and the additional barriers to health care such as culture and language experienced by refugees and asylum-seekers [47], all of which present additional challenges in providing equitable mental health care [48]. We note here though that services involved in refugee mental health care may be seeking to cope not only with elevated risks in refugee populations but also relatively higher morbidity in the rest of their resident populations.

In clinic populations attended by refugees and asylum-seekers, health care assessments should be completed with a high index of suspicion for the presence of co-morbid mental health disorders. It seems important, therefore, that adequate mental health training is provided to primary care providers when delivering care to this group, including training in use of tools such as the K10 and PTSD-8. Our experience suggests that the co-location of mental health services within a broader health setting, and particularly within a specialist refugee health clinic which is skilled at providing culturally-responsive services, is likely to go some way to lowering barriers to early mental health assessment and treatment [47]. Such a service can support pathways for this group to sustainably access universal services in the longer term. At the same time, it is critical as part of planning to build capacity within primary care and specialist mental health services to meet the specific needs of this group.

### Comparisons with literature

Although it is possible to find some consistencies in prevalence rates across selected studies, such comparisons are of limited utility in this highly localised study due to differences in factors such as visa status, recruitment setting, time in host country, ethnicity and measures; large differences in rates of PTSD and depression reported in the literature for refugee populations have been noted previously [4, 5, 49]. Given Syria’s recent rise to become the lead source country for refugees it might be noted, however, that internationally, high rates of mental disorder have been reported in Syrian refugees as well [50, 51]. The greater proportional burden of PTSD (PTSD-8) in the refugee and asylum-seeker group compared to the Australian sample (WHO-CIDI 3.0) is convergent with that reported by Silove et al., [52] who found that, although overall mental health prevalence rates were low in a sample of Vietnamese refugees living in Sydney, 50% of those diagnosed with any mental disorder had PTSD compared to 19% of an Australian comparison group taken from the 1997 NSMHWB.

### Suggestions for future research and comments on methodology

The substantial heterogeneity in prevalence rates in refugee populations underscores the need for both representational community surveying methods as well as local methods when considering need for mental health care. Representational community sampling is important to support policy and service planning at state and national level [4]. It requires a different approach to that undertaken at the single RHS site because the community refugee and asylum-seeker population are members of what have been termed “Hard-to-Reach” groups [53]. Such groups present challenges for survey processes [53], particularly the establishment of a sampling frame that can produce a representative sample of refugees and asylum-seekers residing in the community. The current *Building a New Life in Australia project*: *The Longitudinal Study of Humanitarian Migrants* is an excellent example of representative sampling in the refugee population [54]: reporting findings from this project may add to our knowledge in this area.

Methods of assessing local need are also important, as this may be very different to what is found in large-scale surveys. Thus, while the findings reported here cannot be generalised to the community, even amongst identical cultural groups, they have been important for informing the response of the RHS to the mental health needs of attendees and have contributed to decisions to maintain or extend co-located mental health service provision for instance. The streamlined measures used in this study were administered in a relatively brief interview and acceptability ratings for the interview were high so, with proper validation, there is potential for such measures to be used on a regular basis as part of regular health surveillance to detect need for care and changing patterns over time. The authors can be contacted directly in regards to sharing the translated versions of the instruments with other interested services or researchers.

Although the K10 was not validated in this study it did appear to be a useful screening measure and possibly more useful than the PTSD-8. Similar to Sulaiman-Hill and Thompson [25], who used the K10 with Kurdish and Afghan refugees in Australia and New Zealand, our experience in administering it was that, with the use of suggested elaborations for certain items, it seemed to be readily understood by participants. The good-excellent Cronbach’s alpha values support this impression and are in line with those previously reported in other non-Western [55, 56] and refugee samples [25, 26]. The K10 was well tolerated and did not appear to trigger any negative reactions. It also picked up all screening cases of PTSD but one. Although the PTSD-8 was also well tolerated, if participants did experience distress, it was this instrument that was of concern rather than the K10. Cronbach’s alpha values were also somewhat weaker for the PTSD-8 compared to the K10. It is possible that the K10 alone may be sufficient as a screening tool in this population, with PTSD assessed subsequently on clinical interview. In future research, it will be important to validate this potentially useful tool with a diagnostic instrument across different cultural groups, including establishing clinical cut-off scores. As noted earlier, although the K10 appeared to have good screening properties for PTSD in the RHS sample, this may not apply to other populations where baseline prevalence is lower [44]. The K6, a truncated form of the K10, and the PTSD-8 are the core measures being used to assess mental health status in the *Building a New Life in Australia* project [54] offering the potential to examine further the performance of these measures in a large sample.

The data-matching process, though imperfect, does represent an advance over simply presenting raw prevalence rates and was completed at no additional data-collection cost. Matching a relatively small target sample with over 500 multiply-matched comparators from the NSMHWB enabled some useful comparisons. Some variance in the data were accounted for by matching with age, gender and service use that is associated with different rates of mental illness-for example, people who visit the doctor are more likely to be ill so the results are more likely to accurately reflect sample differences in rates of mental illness. Thus, in relation to the K10, our matching strategy reduced the risk from 3.83 based on all NSMHWB data to 3.16 with matching. This means that we extracted a subset from the NSMHWB that have more mental illness than the overall NHMHWB sample and it excludes the possibility that the higher rate of mental illness in the RHS clinic sample is simply a product of demographics of this population and gives some, more modest, level of correction for service use patterns. Epidemiological studies commonly use matching to control for effects of confounders that are known to influence the outcome of interest [34], in our case age, sex and health service use. Large national data sets are potential sources for drawing unbiased, matched samples in studies so offering an inexpensive and practical method for making some estimation of RR.

## Conclusions

The need for rapid mental health service planning at the local level will continue to be a pressing need globally. Since this study was conducted, Afghanistan has been supplanted by Syria as the lead country of origin for refugees and high levels of mental illness have been observed amongst this population in neighbouring countries. The present large numbers of refugees fleeing war zones puts a demand on mental health services in the receiving countries. The information presented in this study on high absolute and relative risk of mental illness substantiates the increased need for mental health screening and care in this and potentially other refugee clinics and should be considered in relation to local service planning. Providing complex psychotherapy to refugee and asylum-seeker populations in an array of foreign languages is a challenging task requiring a considerable investment in terms of staff training and ongoing service provision. In order to justify such an investment, for example to government or other funding bodies, a first step is having a clear demonstration of need which surveys such as this one described in this paper can provide. This study methodology can be potentially adjusted to accommodate new waves of refugees and changes in dominant languages to enable a rapid method of collecting key information to support service planning. Matching is a useful way to estimate differences between groups, especially when the target group is comparatively small. While there is no intent to generalize the findings, this methodology may be helpful in determining the need for mental health care in other local services in order to assist service planning and to be able to give some indication of the extent of that need relative to a comparison group at no additional cost. Future researchers are encouraged to use the potential numerous comparator groups such as is available in large population surveys. For practical purposes, the K10 has potential as an initial screen for PTSD in this population, though this may not apply outside this setting where the PTSD rate is very high. There is a pressing need to properly validate such instruments for use in both local and large-scale screening of newly arrived refugees in order to both carry out early intervention when needed and to try to prevent worsening of mental health problems.

## Abbreviations

CI: Confidence interval
DSM-IV: Diagnostic and statistical manual of mental disorders, 4th edition, text revision
GPs: General practitioners
HTQ: Harvard trauma questionnaire
IQR: Interquartile range
K10: Kessler psychological distress scale
LR: Likelihood Ratio
NSMHWB: National survey of mental health and well-being
PPV: Positive predictive value
PTSD: Post-traumatic stress disorder
RHS: Refugee health service
RR: Risk ratio
UNHCR: United Nations high commissioner for refugees
US: United States

## References

[1] UNHCR (2015). World at War. UNHCR Global Trends Forced Displacement in 2014.

[2] Resettlement and Other Forms of Legal Admission for Syrian Refugees [https://data2.unhcr.org/en/documents/download/43659]. Accessed 1 Feb 2017

[3] Kirmayer LJ, Narasiah L, Munoz M, Rashid M, Ryder AG, Guzder J (2011). Common mental health problems in immigrants and refugees: general approach in primary care. CMAJ.

[4] Fazel M, Wheeler J, Danesh J (2005). Prevalence of serious mental disorder in 7000 refugees resettled in western countries: a systematic review. Lancet.

[5] Lindert J, Ehrenstein OS, Priebe S, Mielck A, Brahler E (2009). Depression and anxiety in labor migrants and refugees--a systematic review and meta-analysis. Soc Sci Med.

[6] Asylum Trends Australia: 2011–12 - Annual Publication [http://www.border.gov.au/Search/Pages/Results.aspx?k=Asylum%20trends%20in%20Australia]. Accessed 11 Nov 2013

[7] The Department of Immigration and Citizenship Annual Report 2012–13 [http://www.border.gov.au/about/reports-publications/reports/annual]. Accessed 11 Nov 2013

[8] Boat Arrivals in Australia since 1976 [http://www.aph.gov.au/About_Parliament/Parliamentary_Departments/Parliamentary_Library/pubs/rp/rp1314/BoatArrivals]. Accessed 11 Nov 2013

[9] Australia’s Offshore Humanitarian Program: 2011–2012 [http://www.border.gov.au/Search/Pages/Results.aspx?k=Australia%27s%20Offshore%20Humanitarian%20Program:%202011–2012]. Accessed 21 Jan 2015

[10] An Evaluation of the Primary Healthcare Needs of Refugees in South East Metropolitan Melbourne. A report by the Southern Academic Primary Care Research Unit to the Refugee Health Research Consortium [http://refugeehealthnetwork.org.au/an-evaluation-of-the-primary-healthcare-needs-of-refugees-in-south-east-metropolitan-melbourne/]. Accessed 9 Mar 2012

[11] Enticott JC, Cheng IH, Russell G, Szwarc J, Braitberg G, Peek A (2015). Emergency department mental health presentations by people born in refugee source countries: an epidemiological logistic regression study in a Medicare Local region in Australia. Aust J Prim Health.

[12] Slade T, Johnston A, Oakley Browne MA, Andrews G, Whiteford H (2009). 2007 National Survey of Mental Health and Wellbeing: methods and key findings. Aust N Z J Psychiatry.

[13] An introduction to matching and its application using SAS [http://www2.sas.com/proceedings/sugi29/208–29.pdf]. Accessed 17 June 2016

[14] Pang D (1999). A relative power table for nested matched case-control studies. Occup Environ Med.

[15] Shawyer F, Enticott JC, Doherty AR, Block AA, Cheng I-H, Wahidi S (2014). A cross-sectional survey of the mental health needs of refugees and aslyum seekers attending a refugee health clinic: a study protocol for using research to inform local service delivery. BMC Psychiatry.

[16] Von Elm E, Altman DG, Egger M, Pocock SJ, Gotzsche PC, Vandenbroucke JP (2007). Strengthening the Reporting of Observational Studies in Epidemiology (STROBE) statement: guidelines for reporting observational studies. BMJ.

[17] Monash Health (2014). Fast Facts 2013–2014: Monash Health.

[18] A Profile of Health and Wellbeing in Greater Dandenong 2013 [http://www.greaterdandenong.com/document/7085/your-wellbeing]. Accessed 3 Dec 2015

[19] The Victorian Refugee and Asylum Seeker Health Action Plan 2014–2018 [http://refugeehealthnetwork.org.au/victorian-refugee-and-asylum-seeker-health-action-plan-2014–2018/]. Accessed 28 Jan 2015

[20] Summary information: numbers of humanitarian & asylum seeker arrivals to Victoria [http://refugeehealthnetwork.org.au/wp…/Arrivals_planning_data_Mar_2013/]. Accessed 25 Nov 2013

[21] Australian Bureau of Statistics (2009). National Survey of Mental Health and Wellbeing: User’s Guide.

[22] Kessler RC, Andrews G, Colpe LJ, Hiripi E, Mroczek DK, Normand SL (2002). Short screening scales to monitor population prevalences and trends in non-specific psychological distress. Psychol Med.

[23] Slade T, Grove R, Burgess P (2011). Kessler Psychological Distress Scale: normative data from the 2007 Australian National Survey of Mental Health and Wellbeing. Aust N Z J Psychiatry.

[24] Australian Mental Health Outcomes and Classification Network. Kessler-10 Training Manual [https://www.google.com.au/?gws_rd=ssl#q=Australian+Mental+Health+Outcomes+and+Classification+Network.+Kessler-10+Training+Manual]. Accessed 17 Jan 2014

[25] Sulaiman-Hill CMR, Thompson SC (2010). Selecting instruments for assessing psychological wellbeing in Afghan and Kurdish refugee groups. BMC Res Notes.

[26] Slewa-Younan S, Mond JM, Bussion E, Melkonian M, Mohammad Y, Dover H (2015). Psychological trauma and help seeking behaviour amongst resettled Iraqi refugees in attending English tuition classes in Australia. Int J Ment Health Syst.

[27] American Psychiatric Association (2000). Diagnostic and Statistical Manual of Mental Disorders.

[28] Andrews G, Peters L (1998). The psychometric properties of the Composite International Diagnostic Interview. Soc Psychiatry Psychiatr Epidemiol.

[29] Hansen M, Andersen TE, Armour C, Elklit A, Palic S, Mackrill T (2010). PTSD-8: A short PTSD inventory. Clin Pract Epidemiol Ment Health.

[30] Mollica R, McDonald L, Massagli M, Silove D (2004). Measuring Trauma, Measuring Torture: Instructions and Guidance on the Utilization of the Harvard Program in Refugee Trauma’s Versions of The Hopkins Symptom Checklist-25 (HSCL-25) and The Harvard Trauma Questionnaire (HTQ).

[31] Kessler RC, Üstün TB (2004). The World Mental Health (WMH) Survey Initiative version of the World Health Organization (WHO) Composite International Diagnostic Interview (CIDI). Int J Methods Psychiatr Res.

[32] Cummings P, McKnight B, Greenland S (2003). Matched cohort methods for injury research. Epidemiol Rev.

[33] Cummings P, McKnight B (2004). Analysis of matched cohort data. Stata J.

[34] Rigby AS, Robinson MB (2000). Statistical methods in epidemiology. IV. Confounding and the matched pairs odds ratio. Disabil Rehabil.

[35] Profile Report [http://www.medicarelocals.gov.au/internet/medicarelocals/publishing.nsf/Content/South+Eastern+Melbourne-statshp#.VZztw_nzphE]. Accessed 4 Feb 2015

[36] Immigration Detention and Community Statistics Summary 31 August 2013 [http://www.border.gov.au/Search/Pages/Results.aspx?k=Immigration%20Detention%20and%20Community%20Statistics%20Summary%2031%20August%202013]. Accessed 4 June 2015

[37] The Political Terror Scale 1976–2015 [http://www.politicalterrorscale.org]. Accessed 10 June 2015

[38] Migliorini C, Enticott J, Callaway L, Moore S, Willer B (2016). Community Integration Questionnaire: outcomes of people with traumatic brain injury and high support needs compared with multiple matched controls. Brain Inj.

[39] Prins M, Meadows G, Bobevski I, Graham A, Verhaak P, van der Meer K (2011). Perceived need for mental health care and barriers to care in the Netherlands and Australia. Soc Psychiatry Psychiatr Epidemiol.

[40] Goodman SH, Lahey BB, Fielding B, Dulcan M, Narrow W, Regier D (1997). Representativeness of clinical samples of youths with mental disorders: a preliminary population-based study. J Abnorm Psychol.

[41] Thurston IB, Curley J, Fields S, Kamboukos D, Rojas A, Phares V (2008). How nonclinical are community samples?. J Community Psychol.

[42] Andrews G, Slade T (2001). Interpreting scores on the Kessler Psychological Distress Scale (K10). Aust N Z J Psychiatry.

[43] Creamer M, Burgess P, McFarlane AC (2001). Post-traumatic stress disorder: findings from the Australian National Survey of Mental Health and Well-being. Psychol Med.

[44] Streiner D, Geddes J (1998). Some useful concepts and terms used in articles about diagnosis. Evid Based Ment Health.

[45] Enticott JC, Meadows GN, Shawyer F, Inder B, Patten S (2016). Mental disorders and distress: associations with demographics, remoteness and socioeconomic deprivation of area of residence across Australia. Aust N Z J Psychiatry.

[46] Meadows GN, Enticott JC, Inder B, Russell G, Gurr R (2015). Better access in mental health care and the failure of the Medicare principle of universality. Med J Aust.

[47] Cheng I-H, Vasi S, Wahidi S, Russell G (2015). Rites of passage: improving refugee access to general practice services. Aust Fam Physician.

[48] Minas H, Kakuma R, Too LS, Vayani H, Orapeleng S, Prasad-Ildes R (2013). Mental Health Research and Evaluation in Multicultural Australia: Developing a Culture of Inclusion.

[49] Steel Z, Chey T, Silove D, Marnane C, Bryant RA, Van Ommeren M (2009). Association of torture and other potentially traumatic events with mental health outcomes among populations exposed to mass conflict and displacement: a systematic review and meta-analysis. JAMA.

[50] Gammouh OS, Al-Smadi AM, Tawalbeh LI, Khoury LS (2015). Chronic diseases, lack of medications, and depression among Syrian refugees in Jordan, 2013–2014. Prev Chronic Dis.

[51] Addressing Regional Mental Health Needs and Gaps in the Context of the Syria Crisis [http://internationalmedicalcorps.org/document.doc?id=526]. Accessed 9 Dec 2015

[52] Silove D, Steel Z, Bauman A, Chey T, McFarlane A (2007). Trauma, PTSD and the longer-term mental health burden amongst Vietnamese refugees. Soc Psychiatry Psychiatr Epidemiol.

[53] Willis GB, Smith TW, Shariff-Marco S, English N (2014). Overview of the special issue on surveying the hard-to-reach. J Off Stat.

[54] De Maio J, Silbert M, Jenkinson R, Smart D (2014). Building a new life in Australia. Introducing the longitudinal study of humanitarian migrants. Fam Matters.

[55] Fassaert T, De Wit MAS, Tuinebreijer WC, Wouters H, Verhoeff AP, Beekman ATF (2009). Psychometric properties of an interviewer-administered version of the Kessler Psychological Distress scale (K10) among Dutch, Moroccan and Turkish respondents. Int J Methods Psychiatr Res.

[56] Tesfaye M, Hanlon C, Wondimagegn D, Alem A (2010). Detecting postnatal common mental disorders in Addis Ababa, Ethiopia: validation of the Edinburgh Posnatal Depression Scale and Kessler Scales. J Affect Disord.

